# Effects of ketamine and propofol on motor evoked potentials elicited by intracranial microstimulation during deep brain stimulation

**DOI:** 10.3389/fnsys.2014.00089

**Published:** 2014-05-23

**Authors:** Havan Furmaga, Hyun-Joo Park, Jessica Cooperrider, Kenneth B. Baker, Matthew Johnson, John T. Gale, Andre G. Machado

**Affiliations:** ^1^Department of Neurosciences, Lerner Research Institute, Cleveland ClinicCleveland, OH, USA; ^2^Center for Neurological Restoration, Department of Neurosurgery, Neurological Institute, Cleveland ClinicCleveland, OH, USA; ^3^Department of Neurology, University of MinnesotaMinneapolis, MN, USA

**Keywords:** ketamine, propofol, excitability, motor evoked potentials, deep brain stimulation

## Abstract

Few preclinical or clinical studies have evaluated the effect of anesthetics on motor evoked potentials (MEPs), either alone or in the presence of conditioning stimuli such as deep brain stimulation (DBS). In this study we evaluated the effects of two commonly used anesthetic agents, propofol and ketamine (KET), on MEPs elicited by intra-cortical microstimulation of the motor cortex in a rodent model with and without DBS of the dentatothalamocortical (DTC) pathway. The effects of propofol anesthesia on MEP amplitudes during DTC DBS were found to be highly dose dependent. Standard, but not high, dose propofol potentiated the facilitatory effects of 30 Hz DTC DBS on MEPs. This facilitation was sustained and phase-dependent indicating that, compared to high dose propofol, standard dose propofol has a beta-band excitatory effect on cortical networks. In contrast, KET anesthetic demonstrated a monotonic relationship with increasing frequencies of stimulation, such that the highest frequency of stimulation resulted in the greatest MEP amplitude. KET also showed phase dependency but less pronounced than standard dose propofol. The results underscore the importance of better understanding the complex effects of anesthetics on cortical networks and exogenous stimuli. Choice of anesthetic agents and dosing may significantly confound or even skew research outcomes, including experimentation in novel DBS indications and paradigms.

## Introduction

Evoked and event-related potentials are frequently used in laboratory and human research as well as clinical practice, and anesthesia has substantial effects on them. It has been reported that motor cortical excitability during transcranial magnetic stimulation (TMS) depends on anesthetics. Intravenous propofol suppresses motor evoked potentials (MEPs) while intravenous ketamine (KET) does not suppress MEPs and may even facilitate MEPs (Kothbauer et al., 1993; Kalkman et al., 1994; Di Lazzaro et al., 2003; Ziemann, 2004). Furthermore, topographic quantitative electroencephalogram (EEG) recordings in healthy adult humans under propofol anesthesia showed an increase in alpha frequency band (8–12 Hz) and beta frequency band (12–30 Hz) power in the frontal region as the sedation level progressed (Gugino et al., 2001). While the effects of some anesthetics upon evoked-potentials measured in routine clinical practice, such as somatosensory potentials, have been studied (Lieberman et al., 2006), there is an overall lack of understanding of how these agents may affect MEPs, especially in combination with conditioning stimuli (Haghighi et al., 1990; Haghighi, 1998; Zandieh et al., 2003; Lieberman et al., 2006).

Understanding the effects of anesthetics on MEPs during conditioning electrical stimulation is critical in order to minimize misinterpretation of data derived from anesthetized preparations, including those aimed towards improving our understanding of the mechanisms underlying deep brain stimulation (DBS) (Vitek, 2002; Lozano and Mahant, 2004; Montgomery and Gale, 2008). The dentatothalamocortical (DTC) pathway has been extensively studied by several groups. Experiments in non-human primates have characterized the electrophysiology of the dentate nucleus, showing resting frequencies between 20 and 50 Hz as well as intermittent bursts associated with movement onset, with intra-burst frequencies of 80 Hz, 180 Hz and 240 Hz (Aumann et al., 1998). Single pulse stimulation of the dentate nucleus has also been shown to elicit corresponding evoked potentials in the cortex. Supra-physiological stimulation of the dentate has been shown to elicit MEPs in a time-locked fashion (Rispal-Padel et al., 1981, 1982). Our group has previously shown that DBS of the DTC pathway can modulate the amplitude of MEPs elicited by intracortical microstimulation (ICMS) in the propofol-anesthetized rodent (Baker et al., 2010). The ability to regulate cortical excitability has potential translational implications, as we have also shown that chronic DBS of the DTC pathway enhances motor recovery post-ischemia in rodents (Machado et al., 2013b). However, as the MEP studies were performed in the anesthetized state, a possible confound related to the anesthetic condition could not be ruled out and needs to be studied further. In addition, propofol is frequently used in the clinical practice of neuroanesthesia for procedures such as DBS and awake craniotomies with intraoperative physiology, further emphasizing the need to characterize its effects on electrophysiology. In this study we evaluated the effects of propofol on ICMS-elicited MEPs in the rat with and without DBS modulation. In addition, we also evaluated the effects of KET, which is frequently used in neuroscience, on the same electrophysiological paradigm. Our goal is to determine how the choice and dose of anesthetic agent influences DBS modulation of MEPs in order to better interpret preclinical and translational research on emerging DBS indications, including those that may be based on regulation of cortical excitability such as epilepsy (Hamani et al., 2002), pain (Machado et al., 2007, 2013a; Plow et al., 2013) and motor rehabilitation (Machado and Baker, 2012).

Our findings suggest that; (a) cortical excitability, as indexed by the amplitude of MEPs, was higher under KET than propofol; (b) MEP amplitude was higher during ON-DBS, compared to OFF-DBS in all experiments; (c) when combined with DTC DBS, KET was associated with increased MEP amplitude at high DBS frequencies; (d) there was an interaction between propofol dose and DBS frequency such that standard-dose propofol, but not higher dose propofol, potentiated the facilitatory effects of 30 Hz DBS on MEP amplitude and; (e) both standard-dose propofol and KET showed phase-dependency between ICMS and DBS pulses, indicating that these anesthetics may, in a dose-dependent fashion, induce oscillatory effects on thalamocortical networks.

## Materials and methods

### Animals

**Figure 1:**
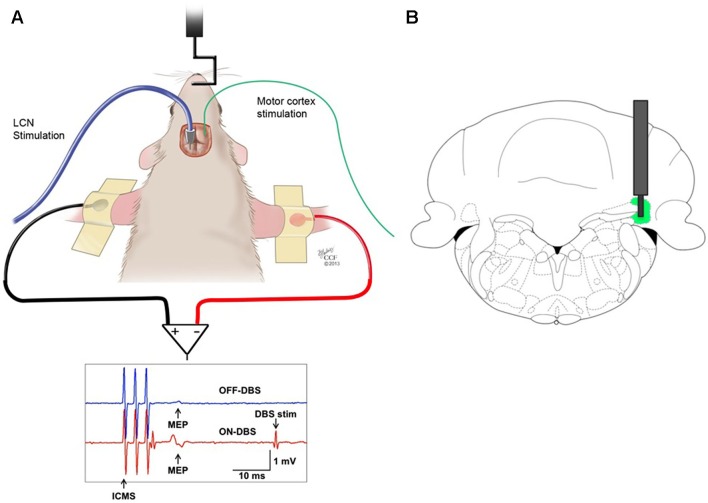
**(A)** Stimulation and recording set-up. MEPs recorded from the biceps brachii muscle in response to ICMS of the contralateral motor cortex before (OFF-DBS panel, first row) and during (ON-DBS panel, second row) stimulation of the LCN, where three pulses ICMS artifacts and STN-DBS artifacts are noticable. **(B)** Illustration of a DBS electrode targeting the LCN, represented in green. The bipolar electrode is shown superimposed on the coronal view of the rat atlas (AP= −11 mm) (Paxinos and Watson, 1998).

All experiments were performed using male Sprague Dawley rats (250–350 g, Harlan, Indianapolis, IN, USA). The animals were housed in an Association for Assessment and Accreditation of Laboratory Animal Care (AAALAC)-approved animal facility in a climate-controlled environment that included a 12-h light/dark cycle and free access to food and water. All procedures were performed under an experimental protocol approved by the Institutional Animal Care and Use Committee (IACUC) and complied with Public Health Service policy. The experimental model utilized ICMS as the test stimulus (Figure 1A). The conditioning stimulus consisted of DBS at the origin of the DTC pathway, in the contralateral lateral cerebellar (dentate) nucleus (LCN) (Figure 1B).

### Cerebellar electrode implantation and experimental procedures

Electrodes were implanted under aseptic conditions as previously described (Machado et al., 2009). Briefly, once anesthetized with a mixture of KET (75 mg/kg) and medetomidine, (0.5 mg/kg) animals were placed in head frame (David Kopf Instruments, Tujunga, CA). A concentric DBS electrode (PlasticsOne, Roanoke, VA) was stereotactically implanted and fixated with the tip targeting the following coordinates relative to bregma: ML = 3.6 mm; AP = −11.0 mm; DV = −6.3 mm (Paxinos and Watson, 1998). A post-operative recovery period of 1 week ensued in order to mitigate potential confounding effects of electrode insertion and to allow for pharmacological washout.

The general procedure for MEP instrumentation and concomitant right, unilateral LCN DBS was conducted as previously described (Baker et al., 2010). For both ICMS and LCN DBS stimulation, stimulation pulses were generated by an isolated electrical current stimulator (STG4008, Multichannel Systems, Reutlingen, Germany). Electromyogram (EMG) was measured with a pair of gold surface disc electrodes (F-E5GH, Grass Technologies, Warwick, RI), filtered with the passing band of 1 Hz–1 kHz, and amplified with an isolated differential amplifier (Octal Bio Amp, ADInstruments, Colorado Springs, CO). After amplification, EMG signals were digitized with 10 kHz sampling frequency and stored to a PC for off-line analysis (PowerLab 16/35 with LabChart Pro, ADInstruments). For the current experiment, animals were assigned to one of three independent groups based upon anesthetic and dose: (1) KET; (2) standard dose propofol (PROP-s); and (3) high dose propofol (PROP-h). All animals received an intravenous line in the tail vein. The loading doses of KET and propofol were 75 mg/kg (i.m.) and 10 mg/kg (i.v.) (Baker et al., 2010), respectively, with no difference in the propofol loading dose regardless of group assignment. Subsequently, KET animals were maintained at a constant infusion rate of 125 µg/kg/min (i.v.) (Adachi et al., 2008), while those in the PROP-s and PROP-h groups were maintained at 400 µg/kg/min (i.v.) (Larsson and Wahlstrom, 1994) or 800 µg/kg/min (i.v.) (Flecknell, 2009), respectively.

Mapping of the motor cortical region for the contralateral forepaw was performed with ICMS through a craniectomy (Barbay et al., 2013) using 50–75 kΩ tungsten microelectrodes (Lot #860841, FHC, Inc., Bowdoin, ME). Figure 1A illustrates the experimental set-up.

### Motor evoked potentials and deep brain stimulation

**Figure 2:**
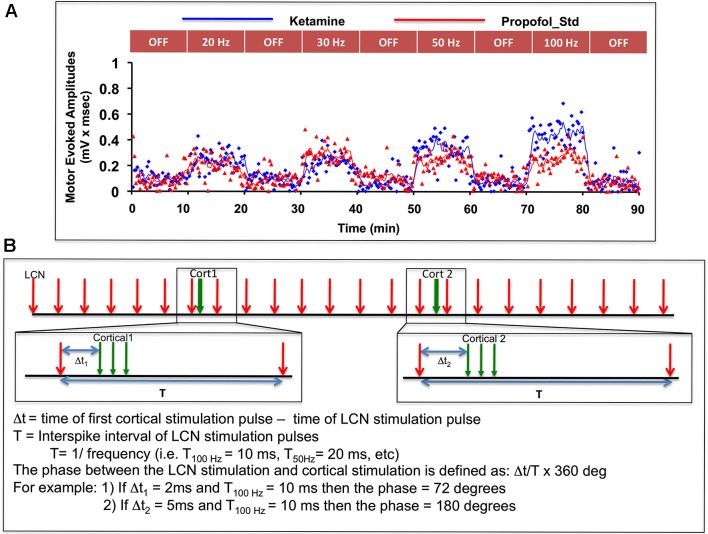
**(A)** Illustration of the experimental sequence for intermittent ICMS and concomitant (but not paired) continuous LCN DBS, with individual MEP data points shown as a function of time for one subject. Only the first 90 min of data are shown in the figure. **(B)** Graphic illustration of the approach used to evaluate possible phase-dependency between LCN DBS pulses and ICMS trains. The phase dependency of MEPs was tested by varying time delay (or phase) between cortical and LCN stimulus pulse. In the bootstrap method, MEPs for each of nine animals were randomly shuffled with respect to the pulse timing and averaged.

Motor thresholds for ICMS and LCN DBS were determined as previously described (Baker et al., 2010). For each animal, LCN DBS was delivered at four specific frequencies (20, 30, 50, and 100 pulses per second), with stimulus amplitude set to 80% of motor threshold. These frequencies were selected based on our previous findings about motor cortical facilitation dependency on LCN DBS frequency (Machado et al., 2009; Baker et al., 2010). Following an initial pre-stimulation 10-min baseline sampling period, LCN DBS was delivered in 10-min blocks, with each ON-DBS block separated from the next by an intervening 10-min DBS-OFF block (Figure 2A). The OFF-DBS epochs were included to identify and characterize any persistent effect (i.e., wash-out period) of LCN DBS on MEP characteristics following cessation of stimulation. In order to minimize any potential order effect, the sequence of DBS frequency blocks was pseudo-randomized across animals.

MEPs were evoked continuously by bursts of ICMS delivered at approximately 15-s intervals (approximately 40 trials per 10-min block) at 125% of motor threshold for forelimb activation. A random interval (± 500 ms) was added to the inter-stimulus interval to minimize the possibility for time-locking between the intracortical burst and the chronic cerebellar stimulation pulses. Each intracortical burst consisted of a train of three charge-balanced square-wave pulses with an intra-burst frequency of 330 pulses per second and a pulse width of 400 µs. Forearm EMG activity was time-locked to the onset of the intracortical burst using stimulus trigger signals. Throughout the experiments, heart rate was continuously recorded as a function of time.

Upon completion, histological examination was conducted as previously described (Machado et al., 2013b).

### Data analysis

Individual MEP responses were reviewed and analyzed using LabChart (ADInstruments, Colorado Springs, CO). Briefly, each MEP response between 8 ms and 17 ms after the first ICMS pulse was rectified and averaged. Then the average rectified EMG prior to stimulation was subtracted to obtain the corresponding MEP magnitude.

The effects of three different anesthetic conditions (KET, PROP-s, and PROP-h) irrespective of time in the OFF-DBS condition were tested with a one-way ANOVA. One-way ANOVA was also used to evaluate the effects of time duration of anesthesia on MEP amplitudes in the OFF-DBS condition for each anesthetic condition separately. The effects of ON-DBS and OFF-DBS under the three anesthetic conditions were analyzed using two-way ANOVA with repeated measures of median MEP values. Two-way ANOVA was also used to evaluate the interaction of the different anesthetic conditions over time on MEP amplitudes. Futhermore, the effects of various DBS frequencies on MEP amplitudes under different anesthetic conditions were analyzed with two-way ANOVA with repeated measures, using median MEPs for each parameter and animal. Statistical significance was accepted at *p* < 0.05 for these analyses.

A bootstrap method was adopted to further examine any potential effect of the relative timing (i.e., phase) of the temporally preceding LCN DBS pulse to the delivery of the ICMS pulses on MEP magnitude (Figure 2B). A total of 1,000 randomizations were performed for each parameter.

## Results

Nine rats per anesthesia group, for a total of twenty-seven rats, underwent LCN DBS electrode implantation and MEP testing. During the OFF-DBS condition, the median MEP amplitude per animal under KET, PROP-s, and PROP-h was 47.5 ± 31.6 µV, 29.3 ± 11.0 µV and 20.4 ± 5.8 µV (mean ± s.d.), respectively. Anesthetics had a statistically significant effect on MEP during OFF-DBS condition (one-way ANOVA, *p* = 0.0225, *F*_(2,24)_ = 4.46), and Bonferroni’s multiple comparison test showed that MEP under KET anesthesia was greater than that under PROP-h (*p* < 0.05), while there was no statistical significance among the other pairwise comparisons.

### ON-DBS MEPs are higher than OFF-DBS MEPs under KET and PROP-s anesthesia

**Figure 3:**
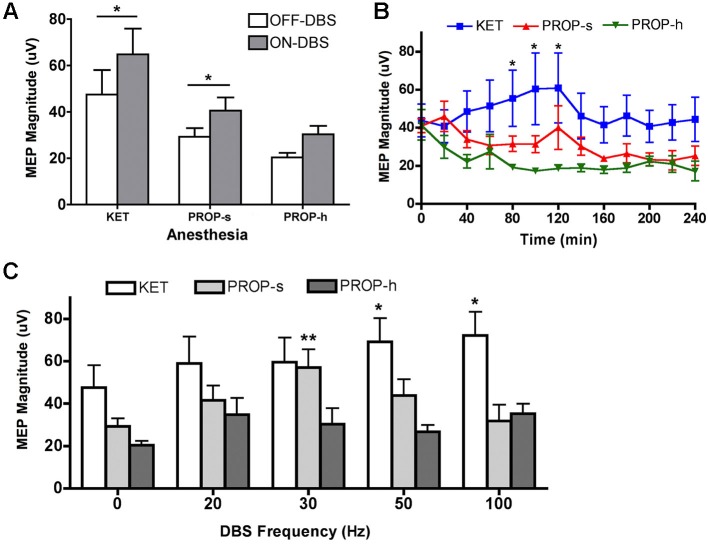
**(A)** MEP magnitudes during OFF-DBS and ON-DBS for different anesthesia methods. Rats were treated with ketamine (KET, 125 µg/kg/min), standard (PROP-s, 400 µg/kg/min) and high (PROP-h, 800 µg/kg/min) dose propofol. *Significant difference between OFF-DBS and ON-DBS (*p* < 0.05, two-way ANOVA, Bonferroni *post hoc*). **(B)** Effects of KET, PROP-s and PROP-h on cortical excitability during OFF-DBS (baseline MEP) over time. *KET is significantly different from PROP-h (*p* < 0.05, two-way ANOVA, Bonferroni *post hoc*). **(C)** Summary of MEP amplitudes modulated by various ON-DBS frequencies under KET or standard or high dose propofol. *^,^**Significant difference from its own baseline (OFF-DBS) condition (*p* < 0.01, two-way ANOVA, Bonferroni *post hoc*).

A general result for this experiment is that, regardless of the pulse frequency of LCN DBS, MEPs were observed to be larger during ON-DBS conditions than in the OFF-DBS condition for both KET and PROP-s anesthetics (Figure 3A). This observation was confirmed using a two-way ANOVA with repeated measures that revealed a significant main effect for anesthesia (*p* = 0.01, *F*_(2,24)_ = 5.545) and DBS (*p* < 0.0001, *F*_(1,24)_ = 25.89) but failed to identify an interaction between anesthesia and DBS (*p* = 0.4604). *Post hoc* analysis demonstrated a significant difference between the ON-DBS and OFF-DBS conditions for both KET (*p* < 0.01 with Bonferroni correction) and PROP-s (*p* < 0.05 with Bonferroni correction).

### OFF-DBS MEP amplitudes under different anesthetic conditions relative to time

To determine the effects of anesthetic agent and dose on cortical excitability over time in the absence of DBS, the amplitude of the MEPs recorded during consecutive OFF-DBS epochs were (Figure 3B). A two-way ANOVA identified a significant effect of anesthesia (*p* < 0.0001, *F*_(2, 259)_ = 22.21), but no main effect for time (*p* = 0.78, *F*_(12, 259)_ = 0.6681). *Post hoc* analysis revealed that MEPs under KET were significantly greater than PROP-h at 80–120 min (*p* < 0.05 with Bonferroni correction). No other significant differences were found between KET and PROP-s or PROP-s and PROP-h.

We further evaluated each anesthetic individually to determine whether its effect on cortical excitability, in the absence of DBS, varied over time by comparing the amplitude of the MEPs recorded across each successive OFF-DBS epoch. Within each anesthetic conditions there were no significant differences in MEP amplitudes over time relative to the initial 10-min OFF-DBS epoch (one way ANOVA, *p* = 0.96). The average median magnitudes of the MEPs recorded at the initial 10-min OFF-DBS epoch were 43.8 µV, 41.0 µV and 41.4 µV for the KET, PROP-s and PROP-h groups, respectively. Although MEP amplitude tended to decrease over time within both PROP-s (*p* = 0.15, *F*_(12, 83)_ = 1.472) and PROP-h (*p* = 0.06, *F*_(12, 73)_ = 1.846) groups, these changes were not significant. The mean reduction in MEP amplitude was 16.8 µV and 24.3 µV, respectively, for PROP-s and PROP-h, over a period of 240 min. In contrast, MEP amplitudes under KET increased over time initially, reaching a maximum of 60.9 µV at 120 min. However, the increase was not significantly different (*p* = 0.98, *F*_(12,103)_ = 0.325) compared to any time point over the 240 min period.

### Anesthetic regimen affects frequency-dependent DBS modulation of MEP amplitudes

To determine the effect of propofol dose on frequency-specific ON-DBS modulation of MEPs, the amplitude of the MEPs recorded under KET, PROP-s and PROP-h were compared. In the two-way ANOVA with repeated measures, the Mauchly’s test of sphericity indicated that the assumption of the sphericity was violated (*χ*^2^(9) = 17.483, *p* = 0.042), and the Greenhouse-Geisser correction (*ε* = 0.753) was used. Two-way ANOVA with repeated measures revealed a significant effect for anesthesia (*p* = 0.01, *F*_(2,24)_ = 5.135), DBS pulse frequency (*p* = 0.002, *F*_(3.01,72.28)_ = 5.539), and the interaction between anesthesia and frequency (*p* = 0.021, *F*_(6.02,72.28)_ = 2.687). In order to understand the relationship between the DBS pulse frequency and the evoked MEP, we performed a *post hoc* analysis independently for each anesthetic regimen comparing OFF-DBS to ON-DBS at 20, 30, 50 or 100 Hz stimulation (Figure 3C). With KET there was a monotonic increase in MEP amplitudes with increased frequencies of stimulation, which was statistically significant at 50 and 100 Hz of stimulation compared to the OFF-DBS condition (*p* < 0.01; with Bonferroni correction). In contrast, PROP-s was associated with an increase in MEP amplitude that peaked at 30 Hz DBS but then returned to near baseline levels (i.e., OFF-DBS) at higher frequencies of stimulation (*p* < 0.001; with Bonferroni correction). Finally, PROP-h was not associated with any significant frequent-dependent modulation of MEP magnitude.

### The facilitatory effects of standard dose propofol and ketamine are phase-dependent

**Figure 4:**
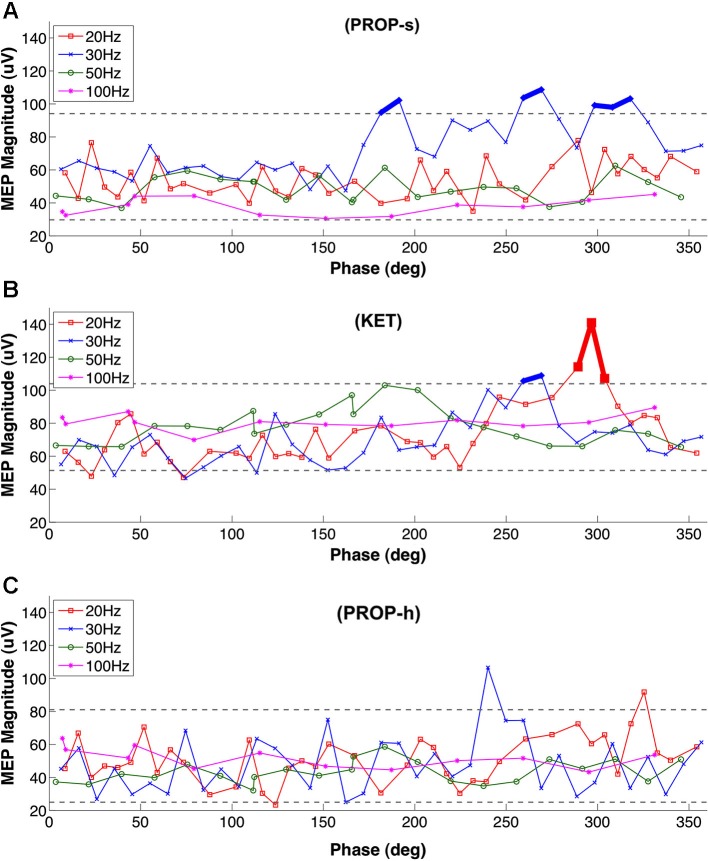
**Phase dependency of MEP under (A) PROP-s and (B) KET (C) PROP-h.** Rats underwent MEP testing at 20 Hz, 30 Hz , 50 Hz or 100 Hz LCN DBS, separated by 10 min washout blocks. The lower and upper gray dashed lines represent 5% and 95% of the bootstrap results, respectively. Two or more MEPs occurring consecutively outside of these lines are considered to be significant and marked with thick lines. For PROP-s, significantly larger MEPs were observed with phase relationships of the ICMS stimuli between 180–190, 270–280, and 310–330 degrees relative to the LCN DBS pulse timing. These correspond to the timing of LCN DBS pulses at 16–17 ms, 25–26 ms and 29–31 ms prior to ICMS, respectively. There were no consecutive MEPs outside of the 5%-95% lines under PROP-h.

The results above point to a filter-like effect associated with standard dose propofol anesthesia, favoring greater MEP amplitudes (and thus cortical excitability) when DBS was set to 30 Hz, but not other frequencies. The findings could be consequent to the potentiation effects between the exogenous 30 Hz stimulation and propofol-induced beta-band paradoxical excitation, previously reported in humans by McCarthy et al. (2008) (see Section Discussion). To examine further the nature of this relationship, we evaluated the effect of 30 Hz LCN DBS pulse timing (i.e., phase) relative to MEP stimulus delivery (Figure 4A). Under PROP-s anesthesia we found that MEPs increased significantly when initiated at phase relationships between 180–190, 270–280, and 310–330 degrees relative to the 30 Hz LCN DBS pulse timing. This finding suggests that under these conditions MEPs are maximized when LCN stimulation is delivered 16–17, 25–26 and 29–31 ms prior to ICMS.

Like PROP-s, we also found a LCN DBS frequency dependency of MEPs under KET and a phase association was also identified. This association occurred at 20 Hz (290–310 degrees, 16–17 ms prior to ICMS) and 30 Hz (260–270 degrees, 22–23 ms prior to ICMS; Figure 4B). No temporal relationship was observed between LCN DBS and ICMS on MEP amplitudes in the PROP-h group (Figure 4C).

## Discussion

In this study we evaluated the effects of propofol and ketamine on cortical excitability in an animal DBS model. Several emerging DBS indications are currently under evaluation, ranging from psychiatric disorders such as depression, obsessive-compulsive disorders and post-traumatic stress disorder to epilepsy. Preclinical investigation in animal models plays an integral role in developing novel therapies and can significantly impact the conduct and design of clinical studies (Hamani and Nobrega, 2012; Hamani et al., 2012; Machado et al., 2013b). Because many rodent studies are conducted in the stereotactic apparatus with the animal under anesthesia, it is important to understand the effects of these anesthetics on electrophysiology (Zandieh et al., 2003; Oria et al., 2008).

In the present study, due to concern for dose-dependent effects related mostly to propofol anesthesia (McCarthy et al., 2008), we selected a single dose of KET (125 µg/kg/min) but two doses of propofol (400 µg/kg/min and 800 µg/kg/min). It is not possible to directly correlate the dose of propofol between humans and rats given the significant metabolic differences across the two species. Hence, we have selected a standard dose of propofol defined as sufficient to maintain the rat under adequate sedation during the experiments with occasional supplemental boluses, and a higher dose, which maintains deep sedation without need for supplemental boluses during the experiment. It is possible that the depth of anesthesia, and thus the effects upon the neural network, presented with some minor variations over time, even though no gross changes were seen on the quality of anesthesia for hypnosis and analgesia. These possible time-dependent variations were controlled by the study design, which compared the magnitude of MEPs during DBS-ON epochs to preceding DBS-OFF epochs, thus reducing any impact of time-dependent anesthetic depth fluctuations on outcome.

We evaluated the effects of specific frequencies on MEP amplitudes and possible interactions with anesthetics. While there were no significant changes in MEP magnitude over time during OFF-DBS epochs among KET-anesthesized animals, a monotonic increase in MEP magnitude with respect to ON-DBS pulse frequency was observed. The mechanisms underlying this frequency dependency under KET are not known but may be mediated by direct excitation of the DTC pathway and increased cortical excitability. This disynaptic pathway is excitatory and stimulation with single pulses at the cerebellar origin has been shown to produce potentials at the thalamus as well as motor cortex (Rispal-Padel et al., 1981; Rispal-Padel et al., 1982). The mechanism of action of KET is rather complex. Moderate doses of KET are thought to increase motor excitability by blocking NMDA receptor-mediated excitatory inputs to inhibitory interneurons (Ziemann, 2004; Brown et al., 2010, 2011). In addition to blocking NMDA receptors, KET also increases the release and inhibits the reuptake of serotonine and norepinephrine (Ziemann, 2004; Zhao and Sun, 2008). Beyond its effects on neurotransmitters, KET has been shown to rapidly increase synaptogenesis in the rat model (Duman et al., 2012). Our ketamine results suggest that the greater temporal summation associated with higher frequency LCN DBS resulted in a net excitatory effect in the motor cortex.

The distinct effects of standard dose versus higher dose propofol on MEP amplitudes are perhaps the most important findings of our study. A significant effect of ON-DBS frequency on MEP amplitudes was noted with standard dose propofol, where MEP amplitude was greatest during 30 Hz LCN DBS. Non-significant increments in amplitudes were also observed at 20 and 50 Hz. In contrast, higher dose propofol was not associated with any significant frequency-dependent changes, suggesting that the use of propofol may be associated with a dose-dependent facilitatory effect that is specific to the upper beta band frequency range. The effects of propofol have been well characterized in modeling studies as well as in human studies utilizing non-invasive (e.g., EEG) techniques during anesthesia (Ching et al., 2010; Purdon et al., 2013). It has been suggested previously that paradoxical beta-band cortical excitation produced by lower doses of propofol are related to the emergence of interneuron antisynchrony secondary to GABA receptor interactions with intrinsic membrane potentials (McCarthy et al., 2008). The paradoxical excitation state is phased out as the depth of anesthesia is increased, from the beta oscillations towards alpha rhythms when there is greater cortical inhibition and ICMS is less likely to elicit a strong motor-evoked response. Our results point to a significant potentiation of effects resulting from the association between standard-dose propofol sedation and 30 Hz DBS. We further investigated this interaction by exploring the existence of possible phase dependence between the cortical test stimuli (that elicited each MEP) and DBS. We found that at 30 Hz ON-DBS, standard dose propofol was associated with the highest amplitudes only when DBS electrical pulses were delivered before ICMS pulses at specific periods. These findings strongly suggest that cortical excitiability is coupled with the exogenous stimulation at certain frequencies under PROP-s. Otherwise, such strong phase dependent potentiation would have not sustained over time. We also examined the possibility of phase association between DBS and MEPs under high-dose propofol and KET. While there was no phase association under higher dose propofol, small windows of phase dependency within the beta frequency range were observed with KET.

Our findings are congruous with previous human studies evaluating the effects of propofol or KET on TMS-elicited MEPs. A detailed review of the effects of various anesthetics on TMS-electited MEPs can be found in Ziemann’s review paper (Ziemann, 2004). Propofol anesthesia was shown to suppress TMS-elicited MEP amplitudes while KET anesthesia had the opposite effect, facilitating MEP responses. In addition to measuring the effects of DBS modulation on MEP amplitude, future studies may also take into account how synchrony in the corticospinal pathway or cortico-thalamic networks influence outcome. In a recent study, Keil et al. demonstrated that coherence between EEG and EMG in the beta-band was linearly correlated with TMS-elicited MEP amplitudes in a time-dependent fashion (Keil et al., 2014).

In summary, our findings stress the importance of understanding the potential effects of different anesthetic agents on experimental DBS and electrophysiological research. These findings can significantly impact the translation of DBS of the DTC pathway for post-stroke rehabilitation as well as other emerging DBS therapies and techniques.

## Conflict of interest statement

Regarding conflict of interest, authors Andre G. Machado and Kenneth B. Baker have potential financial conflict of interest with this research related to intellectual property and distribution rights from intellectual property in IntElect Medical and possible future distribution in Enspire, ATI and Cardionomics. The Cleveland Clinic COI committee has approved a plan for managing the conflict of interest in the conduct of this research. The authors have adhered to the management plan in the conduct and reporting of research findings. None of these entities had any role in the research or preparation of the manuscript. The research was funded by NIH grant R01HD061363.
